# High dose gabapentin does not alter tumor growth in mice but reduces arginase activity and increases superoxide dismutase, IL-6 and MCP-1 levels in Ehrlich ascites

**DOI:** 10.1186/s13104-019-4103-9

**Published:** 2019-01-25

**Authors:** Plinio da Cunha Leal, Ed Carlos Rey Moura, Rachel Jorge Dino Cossetti, Johnny Ramos do Nascimento, Izabel Cristina Portela Bogéa Serra, Bruno de Paulo Ribeiro, Andre Álvares Marques Vale, Ana Paula Silva de Azevedo dos Santos, Flavia Raquel Fernandes do Nascimento, Rioko Kimiko Sakata

**Affiliations:** 1 Federal University of Maranhão, São Luís, MA Brazil; 2 Federal University of São Paulo, São Paulo, SP Brazil; 3 Rua das Boninas, Bloco 2, apartment 1202, condomínio Ile Saint Louis, São Luís, MA 65077-552 Brazil

**Keywords:** Gabapentin, Tumor growth, Cytokine, Arginine, SOD, Ehrlich tumor

## Abstract

**Objectives:**

The purpose of this study was to evaluate the effect of gabapentin on Ehrlich tumor growth in Swiss mice, a highly aggressive and inflammatory tumor model. Mice were grouped into sets of 5 animals and treated from days 2 to 8 with gabapentin 30 mg/kg body weight (G30) or 100 mg/kg body weight (G100), or normal sterile saline (control).

**Results:**

The mice were euthanized on day 10. Tumor growth, tumoricidal agents and inflammatory cytokines levels were assessed. At day 10, G30 and G100 mice gained weight, but there were no differences in tumor cell count or in ascites volume. In G100, there was a reduction in arginase and an increase in SOD activities. There was an increase in IL-6 and MCP-1 levels, especially in G100, but no alterations in TNF-α. There was no direct evidence of tumor induction by gabapentin. However, the findings suggest that its use modulates immune response to a more effector and less deleterious profile, with increase in activity of anti-oxidant enzymes and in cytokines that favor activation of macrophages, which could improve the general status of the tumor host.

**Electronic supplementary material:**

The online version of this article (10.1186/s13104-019-4103-9) contains supplementary material, which is available to authorized users.

## Introduction

Pain is one of the most common and dreaded symptoms in cancer [1]. Guidelines to assist in the management of cancer pain have been developed by the World Health Organization (WHO) more than 20 years ago. They are based on an analgesic ladder, which includes the use of opioid and non-opioid analgesics [2].

Morphine is the most frequently used opioid for the treatment of cancer pain worldwide. However, induction of tumor growth seems to be a potential side effect associated with its use [2]. An interesting option to reduce total opioid dose is the addition of gabapentin. Gabapentin is a first-line drug for the treatment of neuropathic cancer-related pain and a non-opioid adjuvant in the treatment of nociceptive cancer pain [1, 3, 4].

However, the effect of gabapentin in the activity of arginase and nitric oxide (NO) synthase, essential enzymes for normal and malignant cells growth, proliferation and survival, need to be studied to evaluate a possible pro or anti-tumor effect [5, 6].

Bugan et al. [7] suggest possible detrimental effects of higher doses of gabapentin in tumor angiogenesis and growth. In addition, Câmara et al. [8] demonstrated increased inflammatory response and higher cytokine levels associated to gabapentin use in rats submitted to sciatic nerve constriction.

Considering that gabapentin is an important and largely used adjuvant non-opioid drug in cancer pain management [9], and that there are no studies assessing its implications in an inflammatory tumor model, we proposed to evaluate the effects of gabapentin on Ehrlich’s tumor ascites, a model known to create an inflammatory microenvironment that favors for tumor development.

## Main text

### Materials and methods

Fifteen female Swiss mice, 8 weeks of age, weighing on average 28 g, were used. The animals were obtained from the central animal house of the Federal University of Maranhão (UFMA) in São Luís, Brazil, and maintained at 26 ± 2 °C, 44–56% relative humidity, under 12 h light–dark cycles and with free access to sterile food and acidified water. The study was conducted after approval by the Research Ethics Committee for the Use of Animals from UFMA (CEUA 23115.002502/2015-78).

Ehrlich tumor is an aggressive fast-growing breast carcinoma model that leads to ascites and animal death. It is associated to short survival in mice as consequence of higher abdominal pressure and intraperitoneal hemorrhage [10]. This tumor model was selected for the present experiment [11].

Each animal received an intraperitoneal inoculation of 2 × 10^6^ tumor cells. The mice were then divided into three groups of 5 animals to receive gabapentin 30 mg/kg body weight (G30) or 100 mg/kg body weight (G100) daily, or sterile saline solution (control group) from days 2 to 8 (1 week of treatment). Gabapentin or normal saline were diluted in 1% alcohol, and administered orally by gavage once a day. The gabapentin dosage and route of administration have been previously described by Kukkar et al. [12] and Câmara et al. [8], and were selected based on a previous pilot experiment.

The mice were weighed at day 0 and day 10 post-inoculation to assess weight gain. At day 10, the mice were euthanized with an overdose of anesthetic using 150 mg/kg ketamine hydrochloride and 120 mg/kg xylazine hydrochloride. Serum was collected and tumor growth and immunological parameters were evaluated [13, 14].

The abdominal circumference of the mice was measured. The ascitic fluid was collected through an opening in the abdominal wall and careful drainage of all the fluid using a sterile 3 mL syringe. Total ascites volume was measured with a Falcon tube. In sequence, 10 μL of the ascitic fluid was added to 10 μL of Trypan blue dye (0, 2%), and tumor cell count was carried out using a Neubauer chamber [14, 15].

The ascitic fluid was centrifuged (150*g*, 10 min.) and the supernatant was used to evaluate the activity of the inflammatory mediators arginase and superoxide dismutase (SOD) and NO production. Levels of cytokines were measured in serum and ascites (Additional files 1 and 2).

ANOVA test was performed and Student’s *t* test was used to compare the control group to each gabapentin group. Pearson’s coefficient and Spearman test were used for correlations. Graph Pad Prism 5.0^®^ software was used for statistical analysis. p value < 0.05 was considered significant.

### Results

There was weight gain (g) in G30 (11.0 ± 1.3 g; p 0.05) and G100 (10.4 ± 0.7 g; p 0.04) mice compared to the control group (6.8 ± 1.3 g). No differences in abdominal circumference, ascites volume and tumor cell count were observed (Additional file 3: Table S1).

**Fig. 1:**
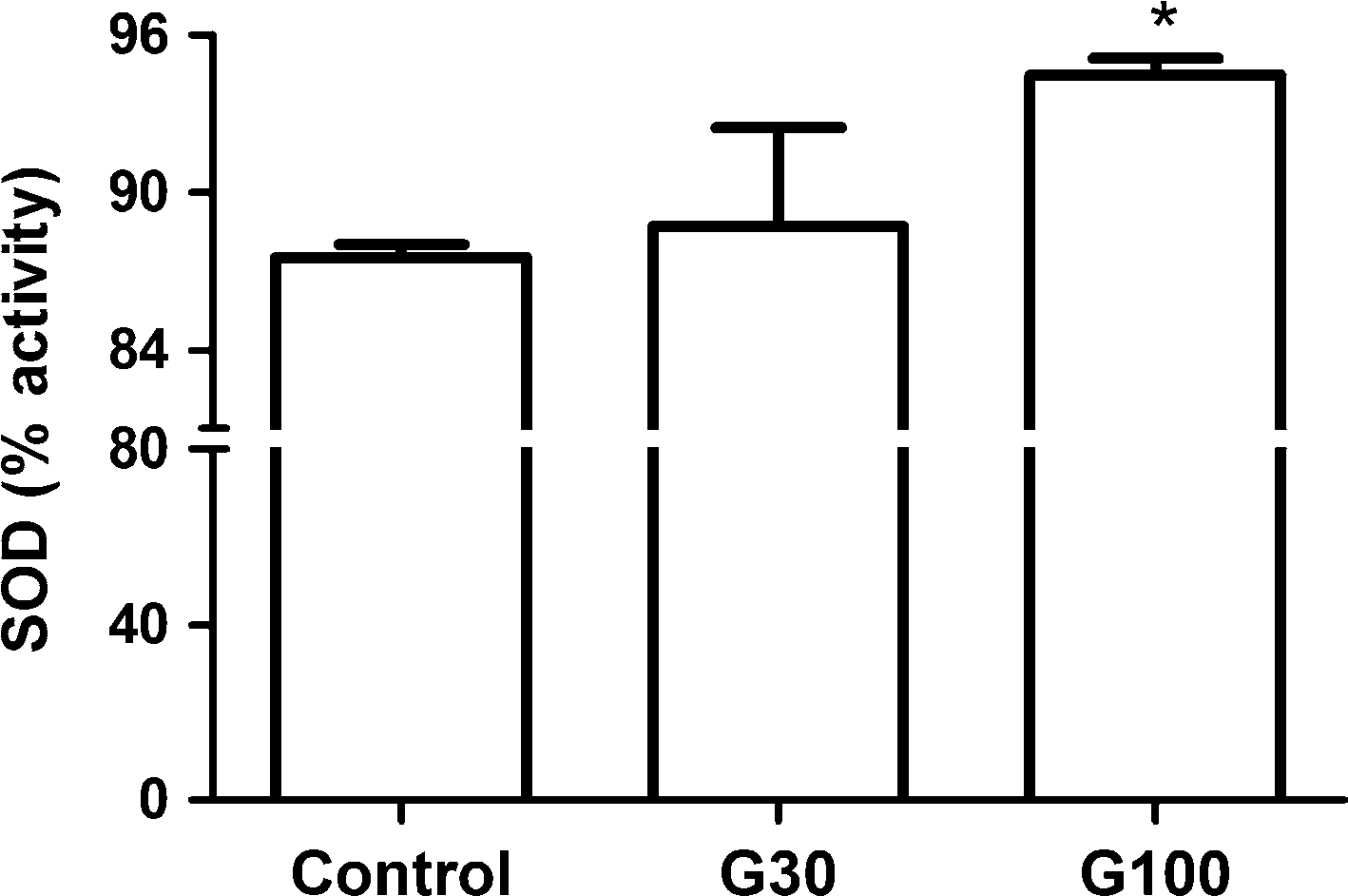
SOD activity in ascites according to study group. Swiss mice received 2 × 10^6^ Ehrlich tumor cells in the peritoneal cavity and were treated with gabapentin (30 and 100 mg/kg) by gavage for 7 days, beginning 24 h after the inoculation. Control mice received saline solution. * p < 0.05 in comparison to control

**Fig. 2:**
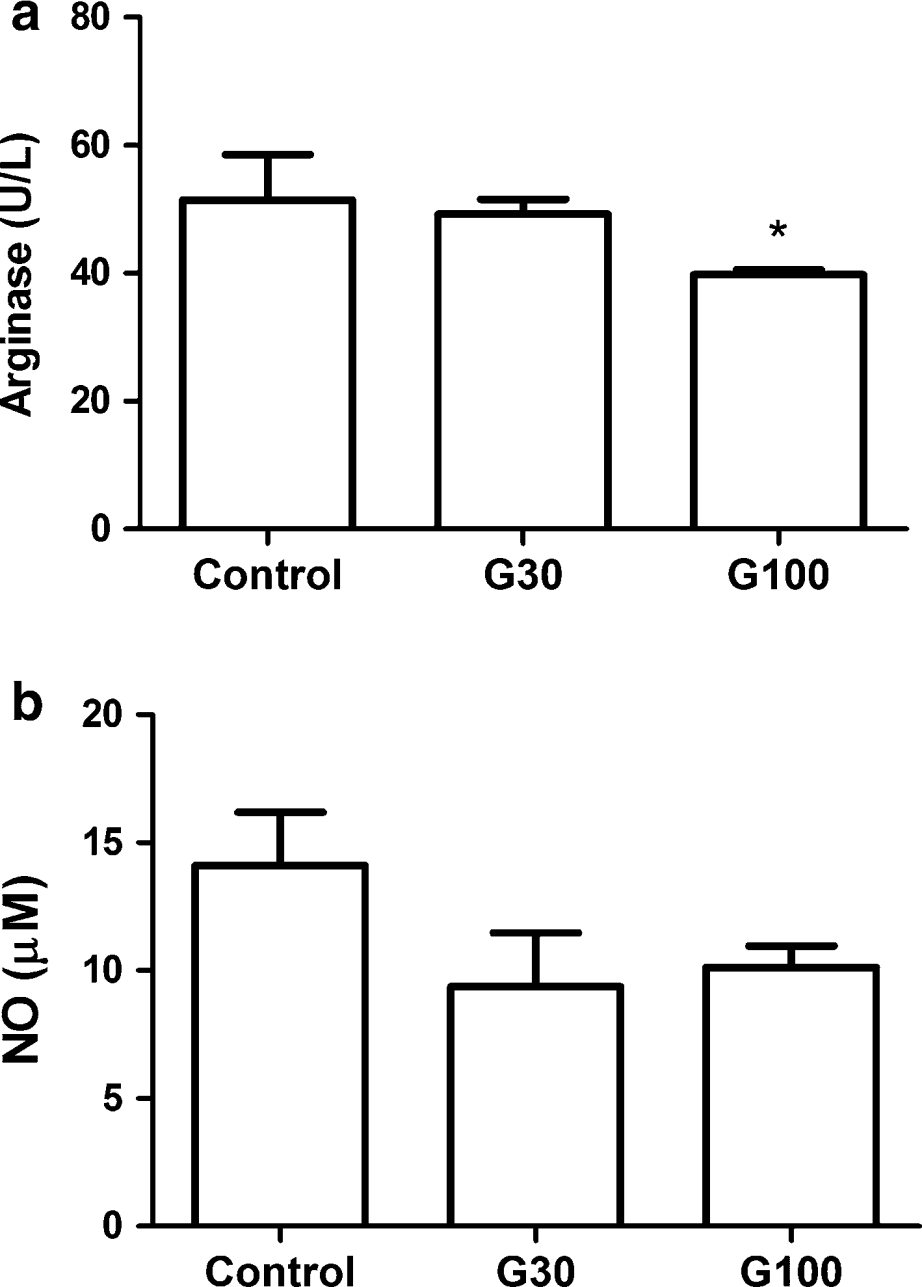
Arginase activity according to study group (**a**) and NO level according to study group (**b**). Swiss mice received 2 × 10^6^ Ehrlich tumor cells in the peritoneal cavity and were treated with gabapentin (30 and 100 mg/kg) by gavage for 7 days, beginning 24 h after the inoculation. Control mice received saline solution. * p < 0.05 in comparison to control

G100 showed an increase in SOD activity levels (p 0.001) and a decreased arginase activity (p 0.001) compared to the control group (Figs. 1 and 2a, Additional file 4: Fig S1). There was no difference in these parameters between G30 and the control group (Fig. 2a) and no difference in NO levels between groups (Fig. 2b).

**Fig. 3:**
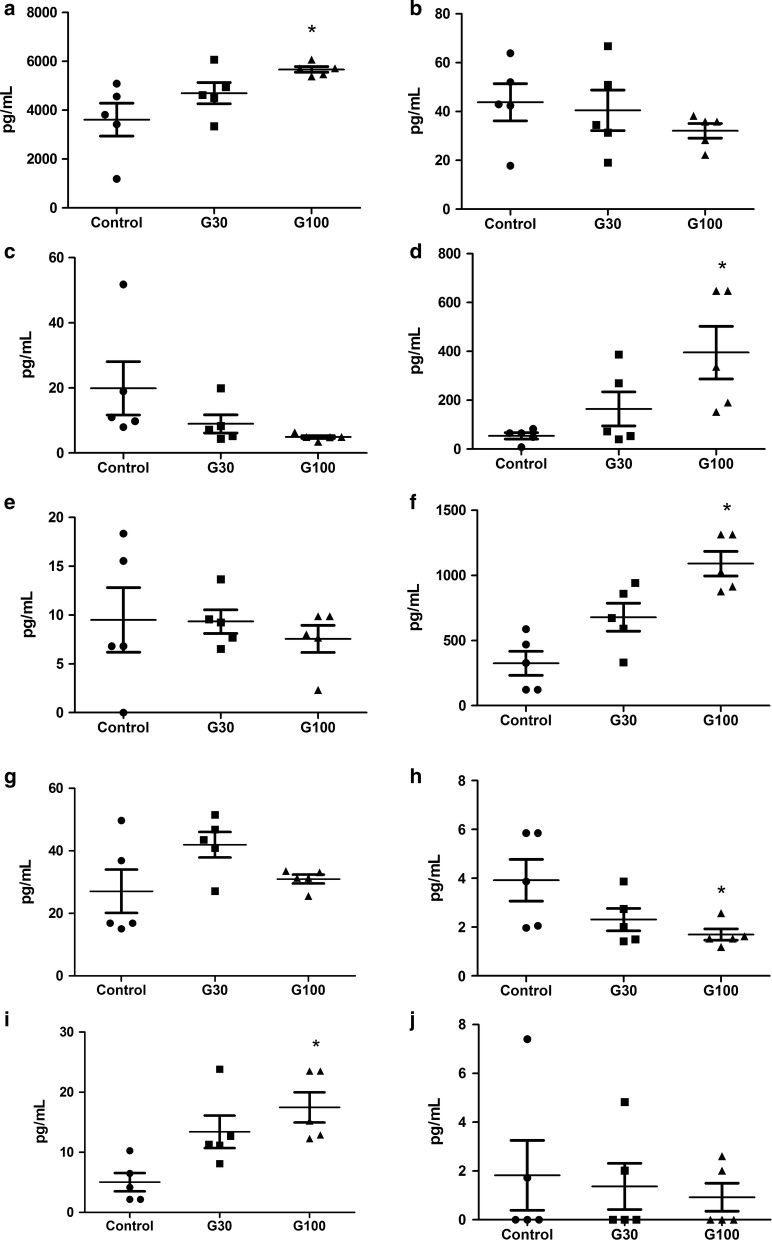
Cytokine level in ascites according to study group: MCP-1 (**a**), TNF- α (**b**), IFN- γ (**c**), IL-6 (**d**) and IL-10 (**e**). Cytokine level in serum according to study group: MCP-1 (**f**), TNF- α (**g**), IFN- γ (**h**) IL-6 (**i**) and IL-10 (**j**). Data expressed as plot by individual mice. * p < 0.05 compared to control

The cytokine profile for each group are shown in Fig. 3. There was an increase in monocyte chemoattractant protein (MCP)-1 levels in ascites (p 0.01) and in serum (p 0.001) and in interleukin (IL)-6 levels in ascites (p 0.01) and in serum (p 0.001) in G100. MCP-1 level was also increased in serum in G30 (p 0.03). Interferon (IFN)-γ level in serum was decreased in G100 (p 0.03), and no difference was seen in G30. Also, there was no difference in IFN-γ level in ascites.

The correlation analysis per mouse showed an increase in the MCP-1 levels in serum and in ascites in G100 (Additional file 5: Fig S2 and Additional file 6: Fig S3). In addition, increased MCP-1 in ascites was associated with elevated SOD activity (Additional file 7: Fig S4).

### Discussion

The current study showed weight gain associated to the use of gabapentin in Ehrlich’s tumor bearing mice. However, it does not seem to be directly associated to tumor growth, since there was no difference in ascites volume, abdominal circumference and tumor cell count.

Gabapentin may be associated with reduced arginase activity and increased SOD activity, as evidenced by the statistically significant difference comparing G100 to control group. Additionally, immunomodulation was observed in G100, as demonstrated by the increase in MCP-1 and IL-6 levels. This suggests that high dose gabapentin induces a repairing inflammation profile, but not a severe inflammatory environment. No previous studies had demonstrated any findings that gabapentin might play a role in inflammation and in immunomodulation in an in vivo tumor model. These findings are relevant because gabapentin is frequently used in the management of severe pain, especially in cancer patients [16, 17].

It is important to emphasize that Ehrlich’s tumor is an extremely aggressive tumor model that creates a microenvironment that favors the development of malignant cells in ascites, with secretion of substances known to promote tumor growth. This provided an appropriate scenario for the evaluation of systemic inflammation in response to the use of gabapentin [10].

This model induces cachexia and increased weight related to ascites volume [18]. However, the weight gain seen in the current study was not associated to ascites volume nor to different food intake (data not shown), suggesting that gabapentin had a beneficial effect in the treated mice.

A study by Bugan et al. [7] demonstrated that the use of a gabapentin dose of 4.6 μg/kg in male Copenhagen rats had no effect in the development of metastasis. However, at a dose of 9.1 μg/kg, the number of lung metastases reduced significantly by 64%, and at an even higher dose of 16.8 μg/kg, there was an increase in metastasis by 112%, with a trend to a shorter mean survival time. Nevertheless, these doses were much lower than in the current study, which were based on previous studies in experimental models [8, 12]. This may justify the paradoxal increase in the number of metastases seen by Bugan et al. with a higher gabapentin dose instead of tumor control, as would be expected by the findings at the intermediate dose.

Arginase and NO synthase take up arginine as substrate, reducing arginine levels. It has been shown that the reduction of arginine levels is associated with tumor progression [19–21]. Also, NO production represents a very important molecular mechanism implicated in gabapentin’s analgesic effects [22]. However, NO synthesis and higher arginase activity are associated to the release of free radicals and tumor growth. Arginase activity causes some important alterations as inhibition of T cell proliferation and activation [23, 24], and antigen-specific T cell responses due to T cell receptor expression inhibition [25]. Therefore, tumor cell count, arginase activity and NO production are correlates of tumor growth and of metabolic pathways that aid in tumor nourishment and growth which are upregulated in several cancers types.

In the present study, these parameters were measured in ascites of Ehrlich’s tumor mice to evaluate the role of the local microenvironment in tumor growth promotion. G100 mice showed reduced arginase activity, but no alteration in NO production. Assuming this would promote an increase in arginine levels, it suggests that gabapentin is beneficial for tumor control.

One of the mechanisms that may be responsible for the reduction of arginase activity is gabapentin-induced calcium channel blockade [5, 6]. Calcium channel blockade is important in gabapentin’s mechanisms of pain control, and it regulates the activation of both NO synthase and arginase enzymes. Thus, calcium channel blockade reduces the activity of substrates that would promote a favorable microenvironment for tumor cells.

Tumor growth has been associated to a reduction of SOD activity [26]. This metalloenzyme has the property of disrupting the superoxide anion by producing hydrogen peroxide, protecting against tumor cell free radicals and oxidative stress [27]. Thus, the higher SOD activity demonstrated in G100 mice suggests a tumor-induced protective mechanism against free radicals, pointing to an anti-oxidant role of gabapentin.

The role of the inflammatory cytokines TNF-α, IL-1β, IFN-γ, MCP-1 and IL-10 in Ehrlich tumor models has been well described [10]. They have been associated to tumor growth and immunomodulation of the inflammatory response. Câmara et al. [8] evaluated the use of three different doses of gabapentin (30, 60 and 120 mg/kg body weight) on Wistar rats undergoing sciatic nerve constriction. At a dose of 60 mg/kg, gabapentin significantly increased nerve myeloperoxidase (MPO), TNF-α, and IL-1β levels; and at 120 mg/kg, there was a reduction in IL-10 level, an anti-inflammatory cytokine. On the contrary, a study by Yamaguchi et al. [28] recent study showed that gabapentin prevented SP-induced IL-6 and IL-8 production in U373 MG cells via the inhibition of signaling molecules, thereby exhibiting anti-neuroinflammatory effects.

In the present study, TNF-α, a cytokine associated to cachexia, was not increased by the use of gabapentin. However, there was an increase in IL-6 and MCP-1 levels in serum and ascites of G100 mice. These cytokines are involved in the attraction and activation of macrophages, which are important for the control of tumor cells (Additional file 5: Fig S2 and Additional File 6: Fig S3). Considering the differences in the experimental models described, it seems that high dose of gabapentin is associated with increased inflammatory cytokines in some experimental models but not in others, as in the tumor model presented here.

In summary, pain stimulus is associated to an inflammatory environment, which interferes with immune mechanisms and promotes tumor growth. The use of the non-opioid adjuvant gabapentin seems to be a good option for the treatment of cancer related pain, with a reduced risk to the oncological patient.

## Limitations

Study results obtained in animals cannot be directly generalized to humans, and they may vary with different tumor types. Future studies are needed to determine if these data can be extrapolated to humans. However, this is the first study to investigate whether gabapentin could have a detrimental effect on tumor cells.

## Additional files

**Additional file 1.** Evaluation of nitric oxide (NO) production in peritoneal cell culture, arginase activity and superoxide dismutase (SOD) activity in ascites fluid and cytokines in serum in ascites fluid.

**Additional file 2.** Datasets used and/or analysed during the current study.

**Additional file 3: Table S1.** Weight gain, abdominal circumference, ascites volume and tumor cell count according to study group.

**Additional file 4: Fig S1.** SOD activity in ascites according to gabapentin dose. SOD activity in ascites increased with higher gabapentin dose.

**Additional file 5: Fig S2.** MCP-1 level in serum according to gabapentin dose. MCP-1 level increased with higher gabapentin dose.

**Additional file 6: Fig S3.** MCP-1 level in ascites according to gabapentin dose. MCP-1 level increased with higher gabapentin dose.

**Additional file 7: Fig S4.** SOD activity according to MCP-1 level in ascites.

## Abbreviations

°C: celsius degree
g: gram
h: hour
IL: interleukin
kg: kilogram
MCP-1: monocyte chemoattractant protein 1
mg: milligram
min: minute
TNF: tumor necrosis factor
WHO: World Health Organization
μg: microgram
μL: microliter
