# Supplementary material for: High dose gabapentin does not alter tumor growth in mice but reduces arginase activity and increases superoxide dismutase, IL-6 and MCP-1 levels in Ehrlich ascites
Source: BMC Res Notes. 2019 Jan 25;12:59. doi: 10.1186/s13104-019-4103-9 (PMC6347815; doi:10.1186/s13104-019-4103-9)
Supplement: Supplementary file 1 — Additional file 1. Evaluation of nitric oxide (NO) production in peritoneal cell culture, arginase activity and superoxide dismutase (SOD) activity in ascites fluid and cytokines in serum in ascites fluid. [file 13104_2019_4103_MOESM1_ESM.docx]

**Evaluation of nitric oxide (NO) production in peritoneal cell culture**

To measure NO, 2x10^6^/mL peritoneal cells were cultured in complete RPMI 1640 medium supplemented with 10 mM HEPES, 11 mM sodium bicarbonate, 100 U/mL penicillin, 100 μg/mL streptomycin, 2 mM L-glutamine, 23 mM L-asparagine, 1mM folic acid, 0.1 mM pyruvic acid and 5% fetal calf serum (FCS) for 48 h at 37ºC in a humid atmosphere containing 5% CO_2_ and 95% air. After the incubation, 50 μL of supernatants were collected and incubated with an equal volume of Griess reagent (1% sulfanilamide/0.1% naphthalene diamine dihydrochloride/2.5% H_3_PO_4_) for 10 min at room temperature, to quantify the accumulation of nitrite. The absorbance was determined at 550 nm. Conversion of absorbance to μM of NO was done by comparing to a standard curve obtained with known concentrations (5-60 μM) of sodium nitrite diluted in RPMI medium [20].

**Evaluation of arginase activity in ascites fluid**

Arginase activity level in ascites (Sigma Aldrich kit, St. Louis, USA), as described by Weisser et al. (2013) [21]. 100μL of ascites samples were added in Amicon Ultra 0.5 mL-MWCO 10KDa centrifugation filters (Merck, Darmstadt, Germany) and centrifuged at 14000g for 1 hour to remove physiological urea. At the end of the centrifugation, the concentrate was recovered and the remaining volume was evenly divided between the test wells and their respective whites. 50 μL/well of the standard urea solution (1 mM) or the samples to be tested (by volume determined after centrifugation) were added in both test wells and white wells. Thereafter, each test well received 10 μL of the arginine-containing buffered substrate and the plate was incubated at 37° for 2 hours to induce the urea formation reaction. After the incubation period, the reaction was stopped by adding 200 μL of the "urea reagent" in all wells, including the standard. The plate was again incubated for 1 hour at room temperature and had its absorbance read at 430 nm. The results were expressed in units/L, where 1 unit of arginase is the amount of enzyme that converts 1 μMol of L-arginine to ornithine and urea per minute at 37°C and pH 9.5 and value expressed as arginase activity (%).

**Evaluation of superoxide dismutase (SOD) activity in ascites fluid**

SOD activity level in ascites was measured following the manufacturer's guidelines and adapted from Andrade et al. (2010). All reagents used for determination of SOD activity were derived from the SOD Assay kit (Sigma-Aldrich, St. Louis, USA). Twenty microlitres of ascites were incubated at 37 °C for 20 minutes with 20 μL of xanthine oxidase solution and 200 μL of WST solution. Absorbance reading was carried out in a spectrophotometer at 450 nm and, after reading the whites, the results were expressed as SOD activity (%), calculated according to the manufacturer's instructions (19160 SOD determination kit).

**Evaluation of cytokines**

The following cytokines were measured in serum and ascites fluid (Fernandes et al., 2015): monocyte chemotactic protein-1 (MCP-1), tumor necrosis factor-α: (TNF- α), interferon-γ (IFN- γ), interleukin (IL)-6 and IL-10. They were determined using a cytometric bead array (CBA) mouse inflammatory cytokine kit for each sample. Three bead populations with different fluorescence intensities detected in the FL3 channel and conjugated to capture antibodies specific for each cytokine were incubated with serum aliquots or a standard in the presence of detection antibodies conjugated to phycoerythrin (PE), which was detected in the FL2 channel. Specifically, 50 µL of serum was supplemented with 50 µL of the bead mixture and 50 µL of the PE detection reagent. The same procedure was repeated for the standard curve. The tubes were homogenized and incubated for 2 h at room temperature in the absence of light according to the manufacturer’s instructions. The samples were run in a FACSCalibur flow cytometer (BD, Franklin Lakes, NJ, USA) and analyzed with CellQuest (BD). The limit of detection of the cytokines are: MCP-1: 52.7 pg/ml; TNF- α: 7.3 pg/ml; IFN- γ: 2.3 pg/ml; IL-6: 5.0 pg/ml; and IL-10: 17.5 pg/ml.
