# Supplementary material for: High dose gabapentin does not alter tumor growth in mice but reduces arginase activity and increases superoxide dismutase, IL-6 and MCP-1 levels in Ehrlich ascites
Source: BMC Res Notes. 2019 Jan 25;12:59. doi: 10.1186/s13104-019-4103-9 (PMC6347815; doi:10.1186/s13104-019-4103-9)
Supplement: Supplementary file 2 — Additional file 2. Datasets used and/or analysed during the current study. [file 13104_2019_4103_MOESM2_ESM.docx]

**Additional file**

**File 1. Effect of gabapentin treatment on weight gain (gram).** Swiss mice received 2x10^6^ Ehrlich tumor cells in the peritoneal cavity and were treated with gabapentin (30 and 100 mg/kg) by gavage for 7 days, beginning 24 hours after the inoculation. Control mice were treated with saline solution.

| WEIGHT GAIN (g) | CONTROL | GABA 30 | GABA 100 |
| --- | --- | --- | --- |
| 1 | 6 | 11 | 12 |
| 2 | 9 | 16 | 9 |
| 3 | 2 | 9 | 10 |
| 4 | 9 | 9 | 9 |
| 5 | 8 | 10 | 12 |

**File 2. Effect of gabapentin treatment on abdominal circumference (cm).** Swiss mice received 2x10^6^ Ehrlich tumor cells in the peritoneal cavity and were treated with gabapentin (30 and 100 mg/kg) by gavage for 7 days, beginning 24 hours after the inoculation. Control mice were treated with saline solution.

| ABDOMINAL CIRCUMFERENCE (cm) | CONTROL | GABA 30 | GABA 100 |
| --- | --- | --- | --- |
| 1 | 10.8 | 10.4 | 10 |
| 2 | 9 | 10 | 10 |
| 3 | 9.5 | 10.5 | 10 |
| 4 | 8 | 10 | 10 |
| 5 | 10 | 10 | 10.5 |

**File 3. Effect of gabapentin treatment on ascites volume (mL).** Swiss mice received 2x10^6^ Ehrlich tumor cells in the peritoneal cavity and were treated with gabapentin (30 and 100 mg/kg) by gavage for 7 days, beginning 24 hours after the inoculation. Control mice were treated with saline solution.

| ASCITES VOLUME (mL) | CONTROL | GABA 30 | GABA 100 |
| --- | --- | --- | --- |
| 1 | 8.12 | 12.5 | 12.4 |
| 2 | 13 | 12.5 | 8.6 |
| 3 | 10.81 | 11.1 | 10.4 |
| 4 | 9.5 | 11.6 | 7.9 |
| 5 | 5.5 | 10.8 | 12.4 |

**File 4. Effect of gabapentin treatment on tumor cell count (x10^7^).** Swiss mice received 2x10^6^ Ehrlich tumor cells in the peritoneal cavity and were treated with gabapentin (30 and 100 mg/kg) by gavage for 7 days, beginning 24 hours after the inoculation. Control mice were treated with saline solution.

| TUMOR CELL COUNT | CONTROL | GABA 30 | GABA 100 |
| --- | --- | --- | --- |
| 1 | 489 | 619 | 726 |
| 2 | 572 | 500 | 522 |
| 3 | 508 | 396 | 410 |
| 4 | 980 | 415 | 336 |
| 5 | 472 | 300 | 336 |

**File 5. Effect of gabapentin treatment on SOD activity in ascites.** Swiss mice received 2x10^6^ Ehrlich tumor cells in the peritoneal cavity and were treated with gabapentin (30 and 100 mg/kg) by gavage for 7 days, beginning 24 hours after the inoculation. Control mice were treated with saline solution.

| ASCITES SOD ACTIVITY (%) | CONTROL | GABA 30 | GABA 100 |
| --- | --- | --- | --- |
| 1 | 87.67 | 89.26 | 92.2 |
| 2 | 87 | 83.63 | 94.81 |
| 3 | 87.63 | 78.59 | 94.24 |
| 4 | 89.15 | 91.05 | 95.59 |
| 5 | 86.15 | 100.94 | 95.58 |

**File 6. Effect of gabapentin treatment on arginase activity in ascites.** Swiss mice received 2x10^6^ Ehrlich tumor cells in the peritoneal cavity and were treated with gabapentin (30 and 100 mg/kg) by gavage for 7 days, beginning 24 hours after the inoculation. Control mice were treated with saline solution.

| ASCITES ARGINASE ACTIVITY (units/L) | CONTROL | GABA 30 | GABA 100 |
| --- | --- | --- | --- |
| 1 | 38.74 | 50.22 | 38.87 |
| 2 | 63.34 | 52.99 | 51.72 |
| 3 | 34.71 | 48.89 | 39.15 |
| 4 | 72.14 | 40.65 | 39 |
| 5 | 48.16 | 53.51 | 42 |

**File 7. Effect of gabapentin treatment on NO level in ascites.** Swiss mice received 2x10^6^ Ehrlich tumor cells in the peritoneal cavity and were treated with gabapentin (30 and 100 mg/kg) by gavage for 7 days, beginning 24 hours after the inoculation. Control mice were treated with saline solution.

| ASCITES  NO (µM) | CONTROL | GABA 30 | GABA 100 |
| --- | --- | --- | --- |
| 1 | 9.02 | 6.04 | 7.8 |
| 2 | 17.56 | 4.28 | 8.96 |
| 3 | 12.52 | 8.76 | 9.86 |
| 4 | 11.18 | 11.48 | 12 |
| 5 | 20.24 | 16.24 | 12 |

**File 8. Effect of gabapentin treatment on MCP-1 level in ascites.** Swiss mice received 2x10^6^ Ehrlich tumor cells in the peritoneal cavity and were treated with gabapentin (30 and 100 mg/kg) by gavage for 7 days, beginning 24 hours after the inoculation. Control mice were treated with saline solution.

| ASCITES MCP-1 (pg/mL) | CONTROL | GABA 30 | GABA 100 |
| --- | --- | --- | --- |
| 1 | 3814.35 | 4626.09 | 5391.1 |
| 2 | 3422.95 | 4504.14 | 6069.08 |
| 3 | 4564.53 | 3343.87 | 5469.62 |
| 4 | 1186.71 | 4952.8 | 5715.44 |
| 5 | 5092.94 | 6069.08 | 5715.44 |

**File 9. Effect of gabapentin treatment on TNF-α level in ascites.** Swiss mice received 2x10^6^ Ehrlich tumor cells in the peritoneal cavity and were treated with gabapentin (30 and 100 mg/kg) by gavage for 7 days, beginning 24 hours after the inoculation. Control mice were treated with saline solution.

| ASCITES  TNF-α (pg/mL) | CONTROL | GABA 30 | GABA 100 |
| --- | --- | --- | --- |
| 1 | 42.97 | 66.75 | 38.33 |
| 2 | 17.81 | 50.9 | 22.21 |
| 3 | 63.89 | 34.49 | 28.37 |
| 4 | 42.44 | 31.34 | 35.9 |
| 5 | 52.1 | 19.11 | 35.9 |

**File 10. Effect of gabapentin treatment on IFN-ϒ level in ascites.** Swiss mice received 2x10^6^ Ehrlich tumor cells in the peritoneal cavity and were treated with gabapentin (30 and 100 mg/kg) by gavage for 7 days, beginning 24 hours after the inoculation. Control mice were treated with saline solution.

| ASCITES IFN−γ (pg/mL) | CONTROL | GABA 30 | GABA 100 |
| --- | --- | --- | --- |
| 1 | 9.78 | 7.13 | 6.34 |
| 2 | 7.98 | 19.89 | 4.87 |
| 3 | 18.91 | 8.28 | 3.45 |
| 4 | 51.8 | 5.23 | 4.94 |
| 5 | 10.97 | 4.39 | 4.94 |

**File 11. Effect of gabapentin treatment on IL-6 level in ascites.** Swiss mice received 2x10^6^ Ehrlich tumor cells in the peritoneal cavity and were treated with gabapentin (30 and 100 mg/kg) by gavage for 7 days, beginning 24 hours after the inoculation. Control mice were treated with saline solution.

| ASCITES  IL-6 (pg/mL) | CONTROL | GABA 30 | GABA 100 |
| --- | --- | --- | --- |
| 1 | 49.55 | 72.29 | 336.93 |
| 2 | 83.3 | 40.19 | 152.4 |
| 3 | 65.91 | 53.99 | 191.17 |
| 4 | 8.58 | 269.88 | 648.47 |
| 5 | 65.91 | 386.9 | 648.47 |

**File 12. Effect of gabapentin treatment on IL-10 level in ascites.** Swiss mice received 2x10^6^ Ehrlich tumor cells in the peritoneal cavity and were treated with gabapentin (30 and 100 mg/kg) by gavage for 7 days, beginning 24 hours after the inoculation. Control mice were treated with saline solution.

| ASCITES  IL-10 (pg/mL) | CONTROL | GABA 30 | GABA 100 |
| --- | --- | --- | --- |
| 1 | 6.81 | 6.52 | 8 |
| 2 | 6.81 | 13.66 | 7.7 |
| 3 | 18.34 | 7.7 | 2.32 |
| 4 | 0 | 9.56 | 9.88 |
| 5 | 15.54 | 9.24 | 9.88 |

**File 13. Effect of gabapentin treatment on MCP-1 level in serum.** Swiss mice received 2x10^6^ Ehrlich tumor cells in the peritoneal cavity and were treated with gabapentin (30 and 100 mg/kg) by gavage for 7 days, beginning 24 hours after the inoculation. Control mice were treated with saline solution.

| SERUM  MCP-1 (pg/mL) | CONTROL | GABA 30 | GABA 100 |
| --- | --- | --- | --- |
| 1 | 327.5 | 591.53 | 877.44 |
| 2 | 587.77 | 673.3 | 1028.11 |
| 3 | 469.44 | 331.4 | 915.52 |
| 4 | 122.96 | 859.16 | 1314.49 |
| 5 | 122.96 | 942.1 | 1314.49 |

**File 14. Effect of gabapentin treatment on TNF-α level in serum.** Swiss mice received 2x10^6^ Ehrlich tumor cells in the peritoneal cavity and were treated with gabapentin (30 and 100 mg/kg) by gavage for 7 days, beginning 24 hours after the inoculation. Control mice were treated with saline solution.

| SERUM  TNF-α (pg/mL) | CONTROL | GABA 30 | GABA 100 |
| --- | --- | --- | --- |
| 1 | 36.86 | 51.5 | 33.12 |
| 2 | 15.06 | 43.51 | 25.57 |
| 3 | 49.71 | 27.14 | 33.57 |
| 4 | 16.87 | 40.87 | 31.34 |
| 5 | 16.87 | 46.82 | 31.34 |

**File 15. Effect of gabapentin treatment on INF-ϒ level in serum.** Swiss mice received 2x10^6^ Ehrlich tumor cells in the peritoneal cavity and were treated with gabapentin (30 and 100 mg/kg) by gavage for 7 days, beginning 24 hours after the inoculation. Control mice were treated with saline solution.

| SERUM  IFN−γ (pg/mL) | CONTROL | GABA 30 | GABA 100 |
| --- | --- | --- | --- |
| 1 | 3.87 | 1.5 | 1.62 |
| 2 | 1.97 | 3.87 | 1.19 |
| 3 | 2.06 | 2.74 | 2.58 |
| 4 | 5.85 | 1.42 | 1.54 |
| 5 | 5.85 | 2.01 | 1.54 |

**File 16. Effect of gabapentin treatment on IL-6 level in serum.** Swiss mice received 2x10^6^ Ehrlich tumor cells in the peritoneal cavity and were treated with gabapentin (30 and 100 mg/kg) by gavage for 7 days, beginning 24 hours after the inoculation. Control mice were treated with saline solution.

| SERUM  IL-6 (pg/mL) | CONTROL | GABA 30 | GABA 100 |
| --- | --- | --- | --- |
| 1 | 10.26 | 8.11 | 12.89 |
| 2 | 6.49 | 12.69 | 12.29 |
| 3 | 4.17 | 11.16 | 15.21 |
| 4 | 2.15 | 11.34 | 23.51 |
| 5 | 2.15 | 23.81 | 23.51 |

**File 17. Effect of gabapentin treatment on IL-10 level in serum.** Swiss mice received 2x10^6^ Ehrlich tumor cells in the peritoneal cavity and were treated with gabapentin (30 and 100 mg/kg) by gavage for 7 days, beginning 24 hours after the inoculation. Control mice were treated with saline solution.

| SERUM  IL-10 (pg/mL) | CONTROL | GABA 30 | GABA 100 |
| --- | --- | --- | --- |
| 1 | 7.4 | 0 | 0 |
| 2 | 1.72 | 0 | 2.02 |
| 3 | 0 | 2.02 | 0 |
| 4 | 0 | 0 | 2.61 |
| 5 | 0 | 4.82 | 0 |
