# Supplementary material for: High dose gabapentin does not alter tumor growth in mice but reduces arginase activity and increases superoxide dismutase, IL-6 and MCP-1 levels in Ehrlich ascites
Source: BMC Res Notes. 2019 Jan 25;12:59. doi: 10.1186/s13104-019-4103-9 (PMC6347815; doi:10.1186/s13104-019-4103-9)
Supplement: Supplementary file 3 — Additional file 3: Table S1. Weight gain, abdominal circumference, ascites volume and tumor cell count according to study group. [file 13104_2019_4103_MOESM3_ESM.docx]

**Additional file 3 Table S1. Weight gain, abdominal circumference, ascites volume and tumor cell count according to study group.**

|  | **Control** | **G30** | **G100** |
| --- | --- | --- | --- |
| Weight gain (g) | 6.8±1.3^a^ | 11.0±1.3 ^*^ | 10.4±0.7 ^*^ |
| Abdominal circunference (cm) | 9.5±0.5 | 10.2±0.1 | 10.1±0.1 |
| Ascites volume (mL) | 9.4±1.3 | 11.7±0.3 | 10.3±0.9 |
| Tumor cell count (x10^8^) | 604.2±95.5 | 446.0±53.7 | 466.0 ±73.4 |

G30: gabapentin 30mg/kg; G100: gabapentin 100mg/kg; control: normal saline

^a^Data represent the mean+ standard error mean (S.E.M.)

*p<0.05 compared to control.
