# Supplementary material for: High dose gabapentin does not alter tumor growth in mice but reduces arginase activity and increases superoxide dismutase, IL-6 and MCP-1 levels in Ehrlich ascites
Source: BMC Res Notes. 2019 Jan 25;12:59. doi: 10.1186/s13104-019-4103-9 (PMC6347815; doi:10.1186/s13104-019-4103-9)
Supplement: Supplementary file 4 — Additional file 4: Fig S1. SOD activity in ascites according to gabapentin dose. SOD activity in ascites increased with higher gabapentin dose. [file 13104_2019_4103_MOESM4_ESM.docx]

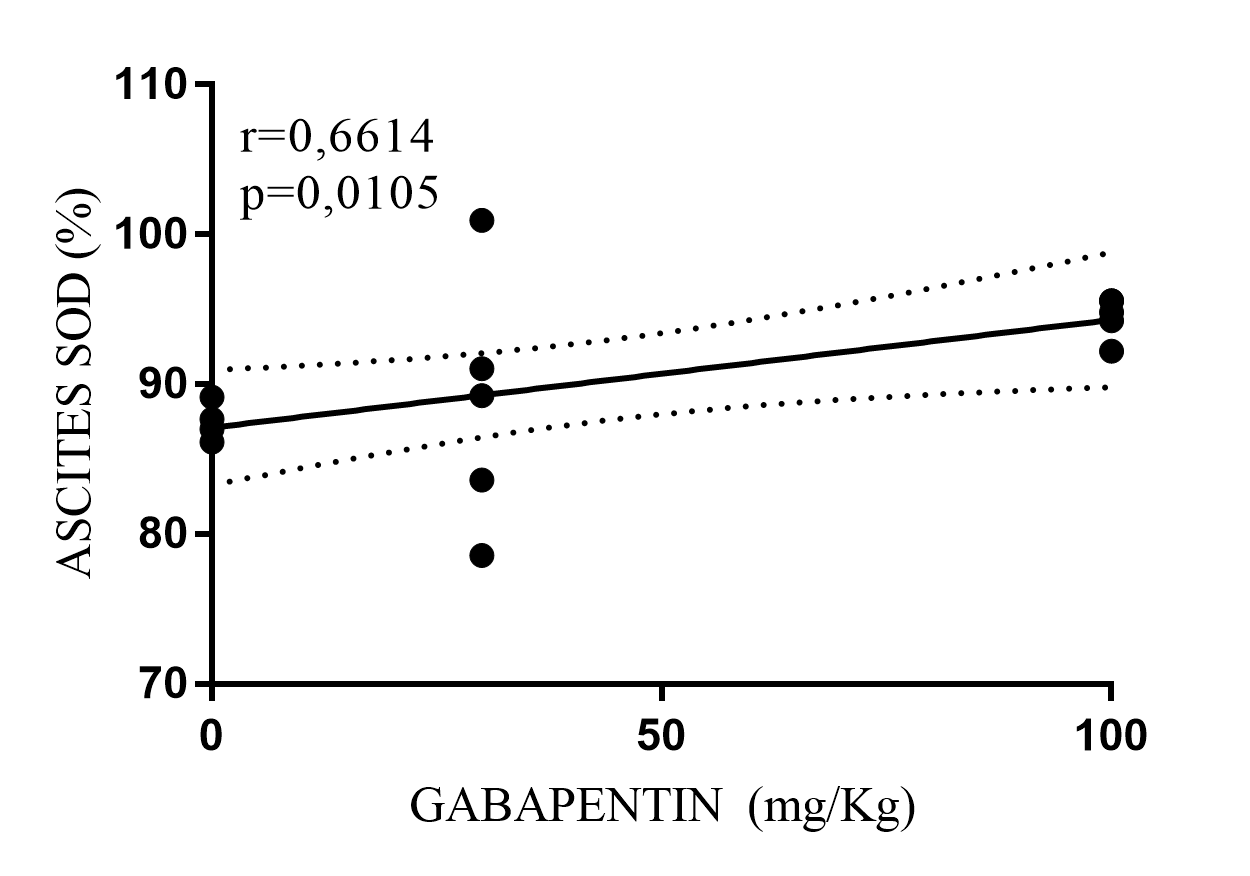


Additional file 4 Fig S1. SOD activity in ascites according to gabapentin dose. SOD activity in ascites increased with higher gabapentin dose.
