# Supplementary material for: High dose gabapentin does not alter tumor growth in mice but reduces arginase activity and increases superoxide dismutase, IL-6 and MCP-1 levels in Ehrlich ascites
Source: BMC Res Notes. 2019 Jan 25;12:59. doi: 10.1186/s13104-019-4103-9 (PMC6347815; doi:10.1186/s13104-019-4103-9)
Supplement: Supplementary file 5 — Additional file 5: Fig S2. MCP-1 level in serum according to gabapentin dose. MCP-1 level increased with higher gabapentin dose. [file 13104_2019_4103_MOESM5_ESM.docx]

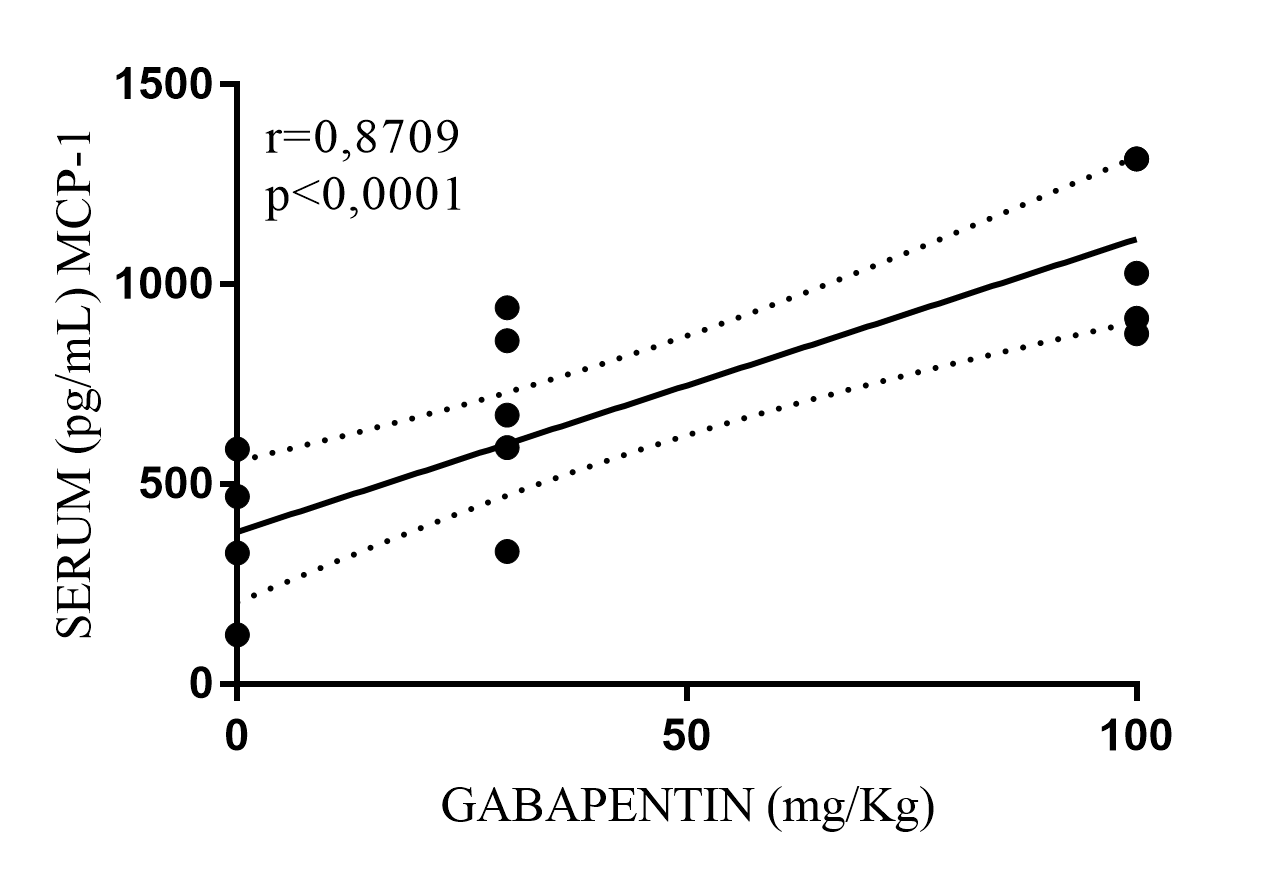


Additional file 5 Fig S2. MCP-1 level in serum according to gabapentin dose. MCP-1 level increased with higher gabapentin dose.
