# Supplementary figures and images for: High dose gabapentin does not alter tumor growth in mice but reduces arginase activity and increases superoxide dismutase, IL-6 and MCP-1 levels in Ehrlich ascites
Source: BMC Res Notes. 2019 Jan 25;12:59. doi: 10.1186/s13104-019-4103-9 (PMC6347815; doi:10.1186/s13104-019-4103-9)

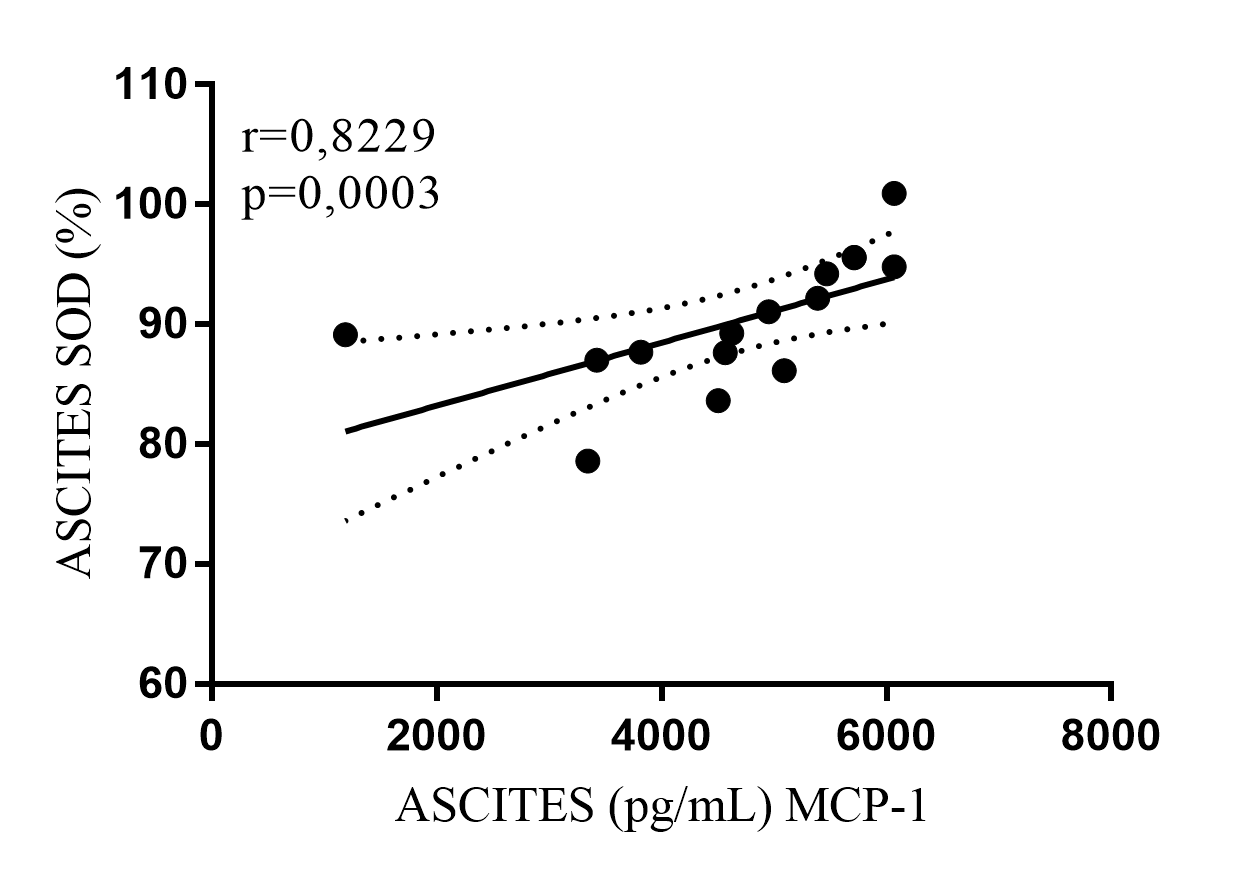


Additional file 7 Fig S4. SOD activity according to MCP-1 level in ascites.

Supplement: Supplementary file 7 — Additional file 7: Fig S4. SOD activity according to MCP-1 level in ascites. [file 13104_2019_4103_MOESM7_ESM.docx]
